# Exploring the Balance between Faradaic and Non-Faradaic Processes in Organic Chemical Reactions at Plasma-Liquid Interfaces

**DOI:** 10.1021/jacs.5c02740

**Published:** 2025-04-12

**Authors:** Casey K. Bloomquist, Daniel Naumov, Ahrin Yang, Ricardo Mathison, Benjamin D. Herzog, William J. Tenn, Miguel A. Modestino, Eray S. Aydil

**Affiliations:** † New York University, Tandon School of Engineering, Department of Chemical and Biomolecular Engineering, 6 Metrotech Center, Brooklyn, New York 11201, United States; ‡ INVISTA, Texas Technology Center, 21920 Merchants Way, Katy, Texas 77450, United States

## Abstract

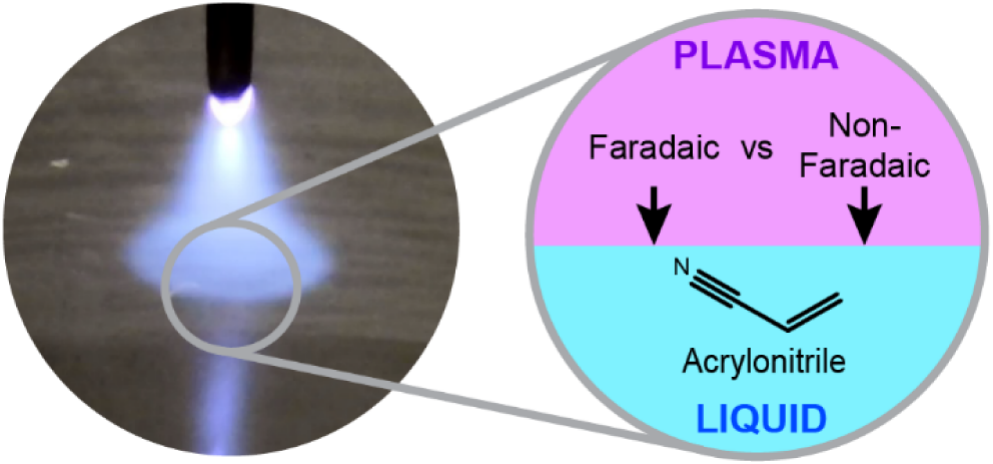

Electrochemistry can enable sustainable chemical manufacturing but is limited by the reactions possible with conventional metal electrodes. Plasma electrochemistry, which replaces a conventional solid electrode with plasma in electrochemical cells, opens new avenues for chemical synthesis by combining Faradaic and non-Faradaic processes at the plasma-liquid interface. To understand how plasma electrochemistry differs from conventional electrochemistry, we investigated plasma reactions with acrylonitrile, an industrially relevant molecule used as the precursor in the well-characterized electrosynthesis of adiponitrile. We demonstrate that non-Faradaic processes dominate plasma-driven chemistry through systematic variation of plasma polarity, current, and reactant concentration, combined with comprehensive quantitative analysis of solid, liquid, and gas products. Most notably, we observed no adiponitrile formation (the desired electrochemical product), while total product yields exceeded the theoretical charge-transfer maximum by up to 32-fold. Substantial polyacrylonitrile formation occurred under all conditions, a product not typically seen in conventional electrochemistry. The plasma anode produced consistently higher yields than the plasma cathode, generating hydrogen and propionitrile at 21 and 2 times the charge-transfer maximum, respectively. Electron scavenger experiments confirmed these transformations occurred primarily through non-Faradaic processes rather than charge transfer. These results demonstrate that plasma electrochemistry with acrylonitrile is primarily driven by non-Faradaic processes at plasma-electrolyte interfaces, providing fundamental insights for harnessing these interactions in chemical synthesis.

## Introduction

Replacing thermochemical processes with electrochemical approaches offers a promising path toward carbon-neutral chemical production.^1−5^ Electrochemical processes are already established at an industrial scale for aluminum production, chloralkali electrolysis, and adiponitrile synthesis for nylon manufacturing. However, conventional electrochemical approaches face fundamental limitations in efficiency and reaction scope due to constraints in catalyst design and the challenge of activating thermodynamically stable molecules. Plasma electrochemistry provides another avenue to leverage electrical energy to drive chemical transformations. While conventional processes rely on solid metal electrodes, plasma electrochemistry replaces one or both^6^ electrodes with an ionized gas. The resulting plasma-liquid interface results in electrochemical reactions where charge transfer between the ionized gas and molecules from the liquid takes place but also enables complex physical and chemical interactions driven by plasma reactive species that drive high-energy chemical reactions otherwise inaccessible in conventional electrolysis.^7,8^ However, the increased reactivity presents challenges as these unique transformations are difficult to control and characterize due to the intricate interplay of electrons, ions, radicals, photons, and physical processes at the plasma-liquid interface.^9−16^

Several recent studies have focused on cathodic plasma electrochemistry applications specifically to harness the large reduction potential of plasma-generated solvated electrons.^17−19^ Researchers have directly measured plasma-generated electrons injected into the liquid phase, where they become solvated,^20^ suggesting that the plasma electrode replaces the metal electrode as an electron source. These solvated electrons not only facilitate charge-transfer reactions analogous to conventional electrochemistry but also extend the 2-dimensional electrode surface into the solution.^21−23^ Experimental demonstrations of solvated electron-driven reductive plasma electrochemistry include nanoparticle synthesis,^12^ ferricyanide reduction,^24^ water electrolysis,^25^ and the reduction of CO_2_ to valuable products such as oxalate and formate.^26^ Cathodic plasma electrochemistry has also shown promise in challenging chemical transformations such as ammonia synthesis^27^ and selective carbon–carbon bond formation.^28^ However, it is well-known that plasma electrolysis involves many non-Faradaic processes in addition to charge transfer.

Early studies of plasma electrochemistry revealed chemical effects that exceeded the yields predicted by Faraday’s laws, which govern charge transfer reactions.^29,30^ To characterize the extent of charge transfer reactions, it is useful to measure production rates in terms of equivalents per Faraday (equiv./F), defined as the moles of product formed per mole of electrons transferred such that yields above 1 equiv./F indicate the presence of non-Faradaic mechanisms (*e.g.,* energy transfer). In one study, observed yields for hydrogen peroxide formation exceeded 1 equiv./F,^31^ while in another, oxidizable species such as ferrous, stannous, cerous, ferrocyanide, and azide ions produced yields greater than 8 equiv./F.^32^ These non-Faradaic yields were attributed to energetic charged particles in the plasma, which are accelerated into the liquid with appreciable kinetic energy, leading to ionization, excitation, and dissociation of solvent molecules, alongside charge-transfer reactions. The resulting reaction mixture contains various radicals and reactive species participating in subsequent reactions. In aqueous solutions, plasma-water interactions primarily generate two key species: hydroxyl radicals (•OH) and hydrogen radicals (H•). While hydroxyl radicals serve as powerful oxidizing agents, hydrogen radicals act as reactive free radicals, both contributing to the unique chemical environment of plasma electrochemistry. This understanding led researchers to conclude that plasma electrochemistry shares more fundamental similarities with radiation chemistry than conventional electrochemistry. While conventional electrochemistry relies primarily on interfacial charge transfer, both plasma electrochemistry and radiation chemistry involve high-energy processes that generate reactive radicals in the solution, initiating cascading reactions.

Recent developments have focused on unraveling the complex mechanisms in plasma electrochemical transformations. For example, researchers demonstrated two parallel mechanisms in hydrogen gas evolution: faradaic liquid-phase reactions via solvated electron reduction of hydronium ions and nonfaradaic gas-phase reactions through electron impact dissociation of water vapor.^33^ Building on this, further studies used pH measurements to distinguish between electrolytic and plasma neutral reactions, revealing that electron transfer dominates in argon and oxygen gas environments while plasma-neutral reactions prevail in nitrogen-containing atmospheres.^34^ Additionally, systematic investigations have advanced our understanding of the contribution of various plasma species and processes on redox chemistry^35,36^ and the mechanisms underlying the degradation of formate and perfluorooctanoic acid (PFOA).^37,38^ Complementing these experimental approaches, modeling efforts have shed light on the structure of the plasma-liquid interface, plasma kinetics, and reactive species densities and fluxes.^21,39−41^ Despite these advances, fundamental questions remain about the balance between Faradaic and non-Faradaic processes in plasma electrochemistry,^42,43^ particularly for organic reactions with multiple reaction pathways controlling their selectivity.

In this study, we advance the understanding of Faradaic and non-Faradaic processes in plasma electro-organic reactions by using a DC pin-to-liquid argon plasma to investigate the chemical transformations of acrylonitrile in aqueous electrolytes. We selected acrylonitrile (AN) as a model reactant, given its industrial relevance in the electrosynthesis of adiponitrile (ADN), a crucial precursor for Nylon-6,6. The electrochemical manufacturing of ADN from AN is one of the largest industrial organic electrosynthesis processes and is well characterized under conventional electrochemical conditions. By comparing this known organic electrochemical reaction to its analog in a plasma electrochemistry configuration, we develop a better understanding of the various processes driving chemical transformations at the plasma-liquid interface and provide quantitative insights into the fundamental differences between conventional electrochemistry and plasma-driven chemistry. Leveraging the ability to operate the plasma as either cathode or anode, we investigated how plasma polarity influences reaction pathways and product distributions. Through a systematic variation of plasma polarity, current, and reactant concentration and a comprehensive analysis of solid, liquid, and gas products, we demonstrate that product yields significantly exceed expectations based on charge transfer alone, highlighting the magnitude of non-Faradaic processes. Based on these results, we highlight potential reaction pathways that account for non-Faradaic processes at the plasma-liquid interface. These findings suggest that charge transfer processes play a limited role in plasma-driven organic chemical reactions of acrylonitrile and that non-Faradaic effects are dominant.

## Results and Discussion

### From Conventional Electrosynthesis to Plasma-Driven Solution Chemistry

**Figure 1:**
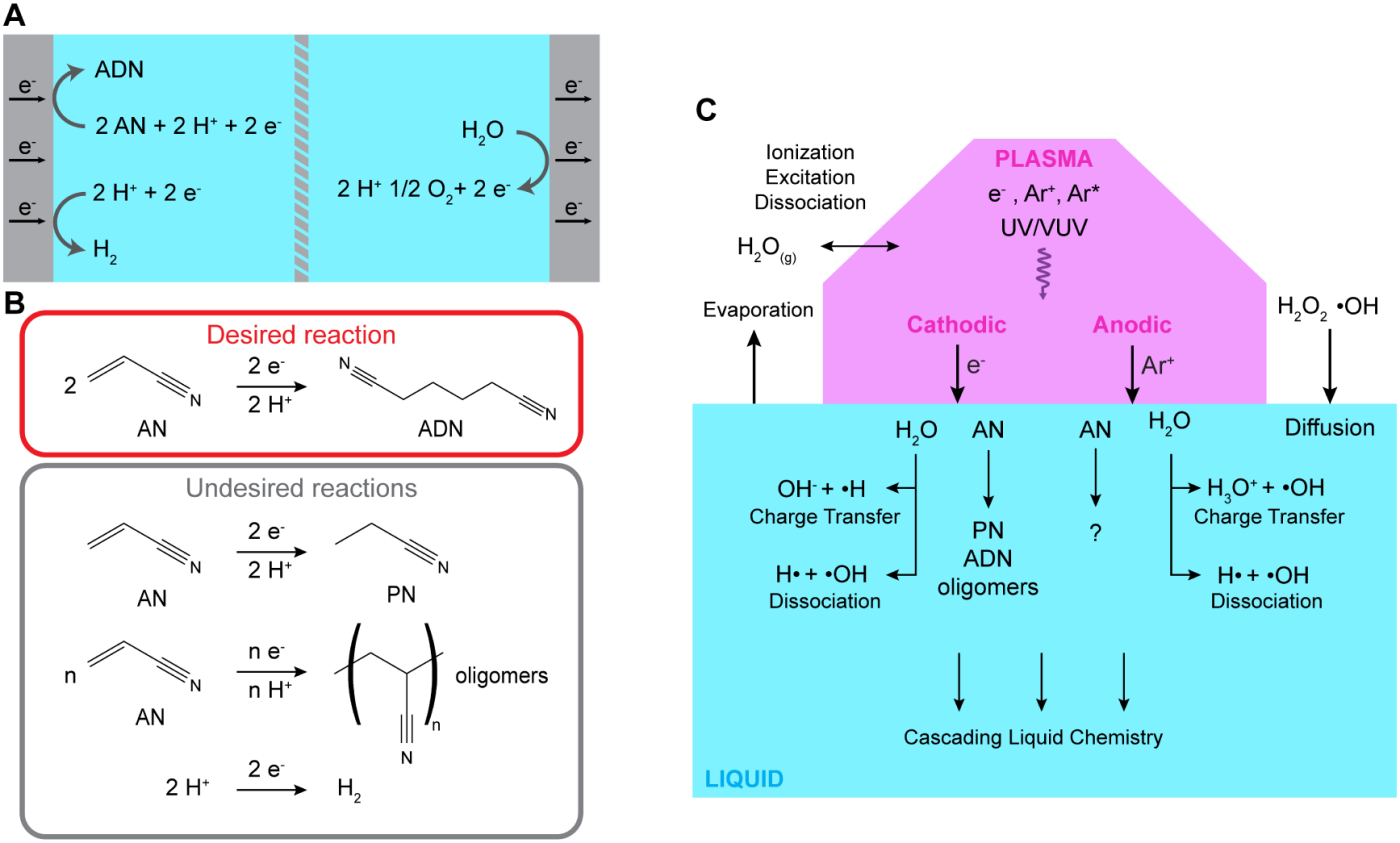
Comparison of conventional electrochemical and plasma-driven reactions of acrylonitrile (AN). (A) Conventional electrochemical processes. In conventional electrosynthesis, adiponitrile (ADN) is the desired product produced at the cathode. With aqueous electrolytes, hydrogen evolution occurs at the cathode, coupled with oxygen evolution at the anode. (B) Cathodic desired and undesired reactions in AN electrosynthesis. Competing reactions include the production of propionitrile (PN), AN-derived oligomers and hydrogen evolution. (C) Plasma electrochemistry enables both conventional Faradaic reactions and unique non-Faradaic pathways. Charge transfer reactions occur due to the charged species injected into the liquid. When the plasma acts as a cathode, electrons (e^–^) are injected into the liquid and initiate reduction reactions whereas when the plasma acts as an anode, positive argon ions (Ar^+^) are injected and induce oxidation reactions. In addition to charge transfer reactions, the energetic plasma species (e^–^, Ar^+^), and excited neutral species lead to energy transfer reactions. In the liquid, this includes dissociation and other non-Faradaic reactions with water, and unknown reactions with AN. Evaporation from the liquid leads to solvent and reactant molecules in the gas phase which can also interact with energetic plasma species. The resulting reactants and reactive species can remain in the gas phase or diffuse into the solution.

The conventional electrochemical synthesis of adiponitrile (ADN) relies on solid electrodes where acrylonitrile (AN) undergoes reductive hydrodimerization at the cathode surface to form ADN (Figure 1A). This process, pioneered and commercialized by Monsanto, has been extensively studied and documented in the literature.^44−49^ Since the reaction is performed in aqueous electrolytes, it competes with hydrogen evolution at the cathode while coupled with oxygen evolution at the anode. As shown in Figure 1B, several side reactions accompany the desired ADN formation, including hydrogen evolution and the production of organic byproducts, particularly propionitrile (PN) and AN-derived oligomers. The distribution of products is largely governed by mass transport conditions: PN and H_2_ formation dominate under mass transport limitations when the local AN concentration is low, ADN production prevails when mass transport and reaction rates are balanced, and larger products (trimers, oligomers, and polymers) form when the AN concentration is high at the electrode surface.

The transition from conventional to plasma electrochemistry involves replacing one metal electrode with a plasma, effectively creating a gas electrode, shown in Figure 1C. In our study, the plasma is formed in argon gas, which primarily produces electrons (e^–^), ions (Ar^+^), and metastable argon species (Ar*). As a first approximation, the plasma can be treated as a source of charged species (electrons and positively charged gas ions) that enable Faradaic reactions through charge transfer at the plasma-liquid interface or within the liquid. The polarity of the applied potential difference determines whether the plasma acts as a cathode (injecting electrons) or an anode (injecting positive ions). When the plasma acts as a cathode (negative polarity), electrons are injected into the liquid phase where they become solvated electrons (e^–^_aq_). These solvated electrons are highly reactive reducing agents that can directly react with water to produce hydroxide ions (OH^–^), hydrogen radicals (H•), and eventually hydrogen gas (H_2_) via second-order recombination.^20,33,43^ The presence of these solvated electrons is a distinctive feature of plasma electrochemistry compared to conventional electrochemical systems. When the plasma is the anode (positive polarity), the plasma injects positive ions, producing H_3_O^+^ and •OH, which can recombine to form H_2_O_2_. In parallel, reactant species undergo reduction (plasma cathode)^12,24,25,43^ or oxidation (plasma anode).^50,51^ This simplified view implies Faradaic product yields that are limited by the total charge transferred. However, additional reaction pathways beyond charge transfer must be considered.

Beyond inducing Faradaic reactions, the charged species (e^–^ and Ar^+^) also impinge on the surface with substantial kinetic energy. For the plasma cathode, electrons are accelerated by the smaller anode fall at the liquid surface with estimated energies <10 eV.^52^ For the plasma anode, the large cathode fall at the liquid surface accelerates Ar^+^ ions to bombard the surface with significant energy (estimates range from 10 to 100 eV).^30,53^ These energetic species transfer their energy, gained from the electric field in the sheath, to molecules near the liquid surface, initiating various energy transfer reactions such as ionization, excitation, and dissociation. In water, this leads to the formation of H•, •OH, H_3_O^+^, OH^–^, H_2_, O_2_, and H_2_O_2_, while organic reactants may undergo parallel energy transfer processes, leading to the cleavage of chemical bonds and molecular reorganizations. These processes also enable gas-phase interactions between plasma species (e^–^, Ar^+^, Ar*) and evaporated species. For water vapor, these include ionization, excitation, and dissociation, primarily generating H_2_O_2_, •OH, H_3_O^+^, and OH^–^, which can diffuse into the liquid phase. Similar gas-phase interactions can occur with evaporated organic molecules, leading to additional reaction pathways. Finally, light emission from the plasma may induce photochemical processes at the interface, generating excited states and initiating photolysis reactions.^38,54,55^ The primary UV emission from our system comes from molecular bands of OH at 309 nm. Additionally, there is potential for VUV emission from Ar_2_* excimer generation at 126 nm, although we expect most of this VUV radiation to be absorbed by water vapor. We classify these energy transfer and non-charge transfer mechanisms collectively as non-Faradaic processes, distinguishing them from Faradaic charge transfer processes.

For AN chemistry specifically, these plasma-liquid interactions suggest multiple reaction pathways. Under plasma cathode conditions, plasma-injected solvated electrons could drive the desired AN reduction and dimerization to ADN through Faradaic processes, like conventional electrochemistry. However, the energetic electrons and various radicals could also initiate energy transfer reaction pathways. Similarly, under plasma anode conditions, interactions with energetic Ar^+^ ions and reactive oxygen species could lead to oxidative degradation of AN, but those effects may go beyond charge transfer yields. In both cases, gas-phase reactions between plasma and evaporated AN molecules can generate unique reactive intermediates, resulting in product distributions distinct from conventional electrochemistry. Polymerization could be induced, either through free radical polymerization initiated by plasma-generated radicals (e.g., •OH) or via photopolymerization initiated by UV radiation.^56−58^ The relative importance of these pathways would likely depend on parameters such as plasma polarity, applied current, and AN concentration and will ultimately determine the balance between Faradaic and non-Faradaic reactions.

### Data-Driven Systematic Exploration of Plasma-Electrochemistry Driving Factors

**Figure 2:**
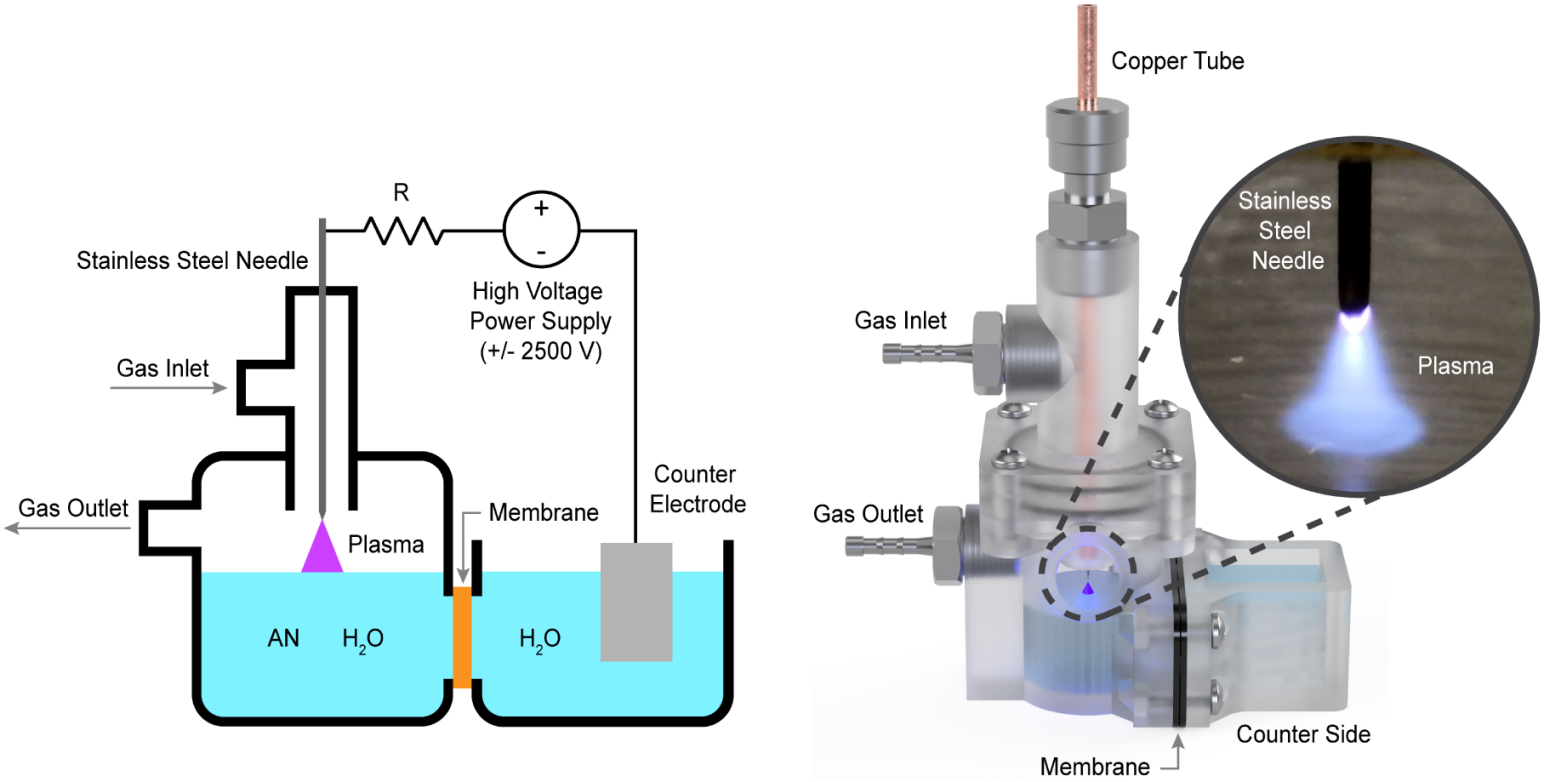
Plasma electrochemical reactor schematic (left) and rendering (right) with a plasma electrode and conventional counter electrode. The plasma is formed in argon gas between a stainless steel needle and the solution surface using a high voltage power supply (±2500 V) and can operate as a plasma cathode or anode. An ion exchange membrane separates the plasma and counter chambers, enabling the collection of only plasma-generated solid, liquid, and gas products from the plasma chamber. The counter reaction chamber was filled with water and performed water electrolysis reactions. The reactor was fabricated with VeroClear Polyjet resin on a Stratasys Objet30 3D printer.

To investigate plasma-electrochemical reactions, we modified a conventional electrochemical H-cell by replacing one metal electrode with a pin-to-plane plasma (Figure 2). The plasma electrode comprises a stainless-steel needle suspended above the liquid surface, and high-voltage DC power supply generates the plasma, which can function as either a cathode or an anode, depending on the polarity of the applied potential. Argon gas flows continuously through the reactor and serves as the plasma medium. An ion exchange membrane separated the plasma chamber from the counter chamber, which contained water and supported water electrolysis reactions.

**Figure 3:**
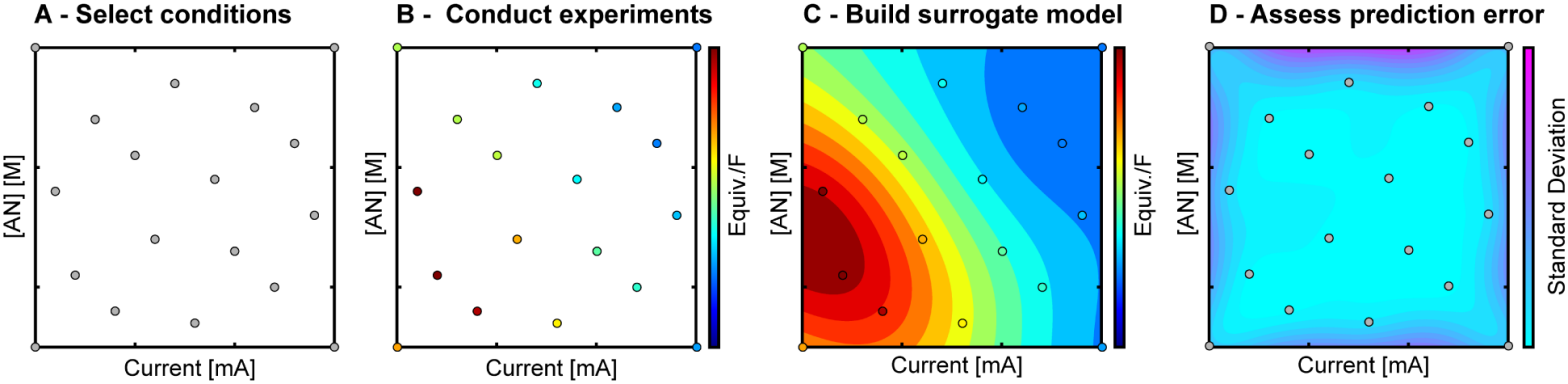
Data-driven surrogate model development workflow. (A) Selection of experimental conditions using Hammersley sampling for pseudorandom distribution. (B) Collection of experimental data from plasma reactions at selected conditions. (C) Development of surrogate models using Gaussian process regression (GPR). (D) Assessment of prediction error.

We conducted a systematic study of plasma polarity (plasma cathode/anode), current, and reactant concentration ([AN]) to investigate the relative contributions of Faradaic and non-Faradaic processes in plasma electrochemistry. Because directly observing individual contributions from various non-Faradaic processes is challenging, our approach (Figure 3) combined systematic experimentation with supervised machine learning to identify trends in product yields relative to experimental parameters and infer the dominant reaction pathways. We selected experimental conditions (Figure 3A) using Hammersley sampling,^59^ a pseudorandom technique ensuring uniform parameter space coverage, with AN concentration from 0 to 0.3 M and currents from 1 to 4 mA. These ranges were chosen to maintain stable plasma conditions and prevent excessive solid formation and carbon buildup that can occur at high initial AN concentration, allowing for measurable product formation while avoiding short-circuiting and preventing excessive polymer decomposition or plasma needle heating. We conducted experiments for both plasma cathode and anode configurations (Figure 3B), identifying and quantifying solid, liquid, and gas products. To analyze the data, we developed surrogate models (Figure 3C) using Gaussian Process Regression (GPR), a machine learning technique that captures complex, nonlinear relationships between input parameters and output variables and their predicted probability distributions.^60−62^ We then used the models to provide a continuous view of product distribution trends across the parameter space after comparing the GPR-provided prediction errors (Figure 3D) with our experimental errors to validate the models.

The products observed in our study revealed fundamental differences between conventional electrochemistry and plasma electrochemistry of acrylonitrile. Below, we present a systematic analysis of the primary products, starting with the hydrogen products expected from water and then moving to organic products to characterize the underlying reaction mechanisms in AN plasma electrochemistry.

**Figure 4:**
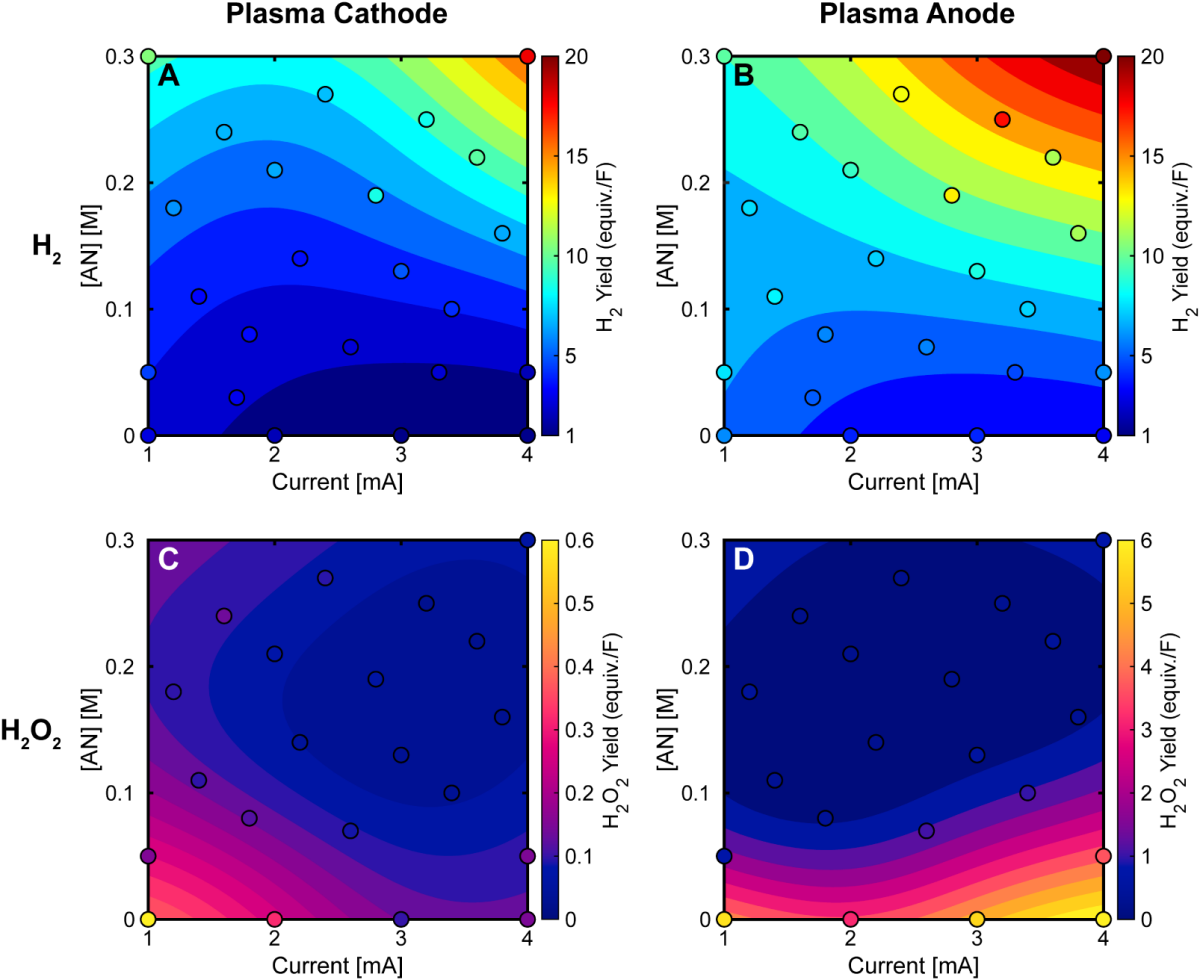
Hydrogen gas (H_2_) and hydrogen peroxide (H_2_O_2_) yields for plasma electrochemical reactions of acrylonitrile (AN) as a function of plasma current, AN concentration ([AN]), and plasma electrode polarity. (A,B) H_2_ yields and (C,D) H_2_O_2_ yields for plasma cathode (A,C) and plasma anode (B,D) configurations, expressed in equivalents per Faraday (equiv./F). H_2_ yields exceed 1 equiv./F even without AN present (0 M) and increase substantially to 15–21 equiv./F with increasing [AN] for both configurations. H_2_O_2_ yields are highest (∼6 equiv./F) for the plasma anode when no AN is present (pure DI water, 0 M AN) and rapidly decrease as AN is added. The yields for the plasma cathode are ∼10 times lower than for the plasma anode across all condition. Surface plots show predicted values from data-driven surrogate models. The reaction solutions consisted of AN in DI water, and the reaction time was 15 min. H_2_ was quantified using gas chromatography (GC) and H_2_O_2_ was quantified using colorimetry.

Hydrogen gas (H_2_) and hydrogen peroxide (H_2_O_2_) yields are shown in Figure 4. In pure water (0 M AN), charge transfer mechanisms predict hydrogen gas (via H• recombination) as the primary reduction product with a plasma cathode, and hydrogen peroxide (via •OH recombination) as the primary oxidation product with a plasma anode. Our results show that while hydrogen gas is indeed produced with the plasma cathode and pure water, the yields exceed 1 equiv./F, consistent with previous findings by Toth et al.^33^ Interestingly, when using a plasma anode with pure water, hydrogen gas yields not only exceed 1 equiv./F but are higher than those observed with the plasma cathode. Hydrogen peroxide production in pure water shows even more striking differences between electrode configurations: while yields remain very low with the plasma cathode, they are substantially higher (>10×) with the plasma anode, exceeding 1 equiv./F and reaching up to 6 equiv./F, aligning with observations by Davies and Hickling^31^ and modeling by Keniley et al.^41^

The addition of AN significantly impacts the production of both hydrogen and hydrogen peroxide. Hydrogen production exceeds Faradaic yields (>1 equiv./F) across all tested currents and concentrations, surpassing the quantities generated by conventional electrochemistry, as shown in Figure 4A,B. As AN concentration increases, hydrogen production rises dramatically, reaching maxima of 16.6 and 21.0 equiv./F for the plasma cathode and anode configurations, respectively, at 4 mA and 0.3 M AN. The relationship between current and hydrogen yield shows a concentration-dependent behavior: at low AN concentrations (<0.1 M), yields decrease slightly with increasing current, while at higher AN concentrations (>0.1 M), yields increase with current. The plasma anode configuration consistently generates 1.25–2 times more hydrogen than the plasma cathode, except at low current (1 mA) and high AN concentration (0.3 M), where yields become equivalent. Meanwhile, hydrogen peroxide production (Figure 4C,D) displays an inverse relationship with AN concentration, with H_2_O_2_ yields rapidly declining as AN concentration increases, though still higher for the plasma anode. This behavior suggests that hydroxyl radicals, which typically combine to form H_2_O_2_, instead preferentially react with AN, or their formation is suppressed by competing reactions of plasma species with AN.

The organic product distribution reveals fundamental differences between conventional and plasma electrochemistry of acrylonitrile. Most notably, adiponitrile (ADN), the primary product in conventional AN electrosynthesis, was not detected under any tested conditions (Figure S6). This absence likely stems from two factors: the limited role of surface-mediated reactions in plasma electrochemistry and insufficient generation of key radical intermediates. While propionitrile (PN), an undesired product in conventional AN electrochemistry, was observed, we also detected substantial formation of polyacrylonitrile (PAN), which is not typically produced in conventional electrochemistry under equivalent conditions. The formation of PAN is likely a result of the free-radical polymerization of AN initiated by radicals in the liquid and/or promoted by UV radiation from the plasma.

**Figure 5:**
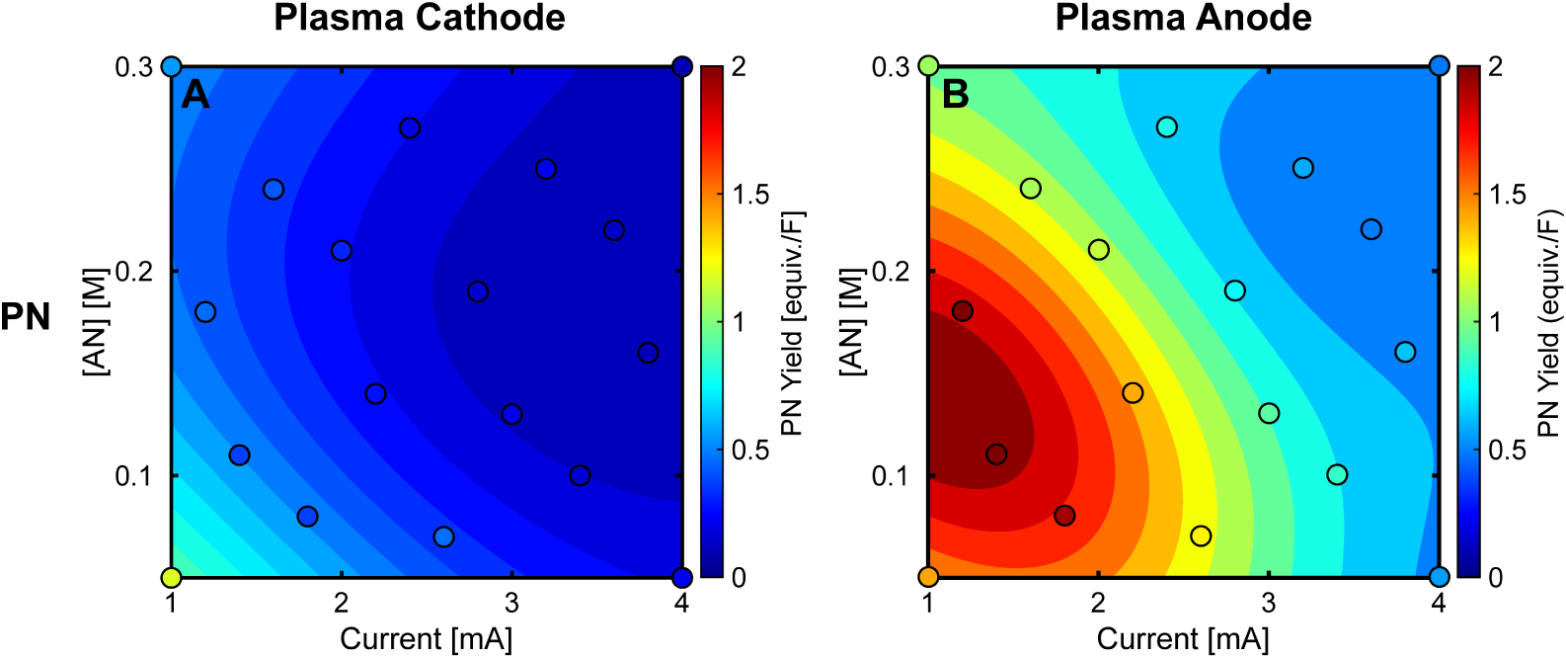
Propionitrile (PN) yields for plasma electrochemical reactions of acrylonitrile (AN) as a function of plasma current, AN concentration ([AN]), and plasma electrode polarity. (A,B) PN yields for plasma cathode (A) and plasma anode (B) configurations, expressed in equivalents per Faraday (equiv./F). PN yields remain below 1 equiv./F for the plasma cathode but exceed this value for the plasma anode, suggesting non-Faradaic mechanisms. Surface plots show predicted values from data-driven surrogate models. The reaction solutions consisted of AN in DI water, and the reaction time was 15 min. PN were quantified using gas chromatography–mass spectrometry (GC-MS).

Propionitrile formation shows distinct behavior between plasma configurations. With the plasma cathode (Figure 5A), PN yields initially appear to align with conventional electrochemical expectations, approaching but not exceeding 1 equiv./F at low current (1 mA) and low AN concentration (0.05 M) and decreasing as either parameter increases. The plasma anode (Figure 5B) produces PN yields that surpass 1 equiv./F, reaching up to 2 equiv./F, despite the absence of a known oxidative pathway from AN to PN. Furthermore, the plasma anode consistently generates higher PN yields, averaging approximately 4 times greater than the plasma cathode across all conditions (ranging from 1.7× to 5.8×).

**Figure 6:**
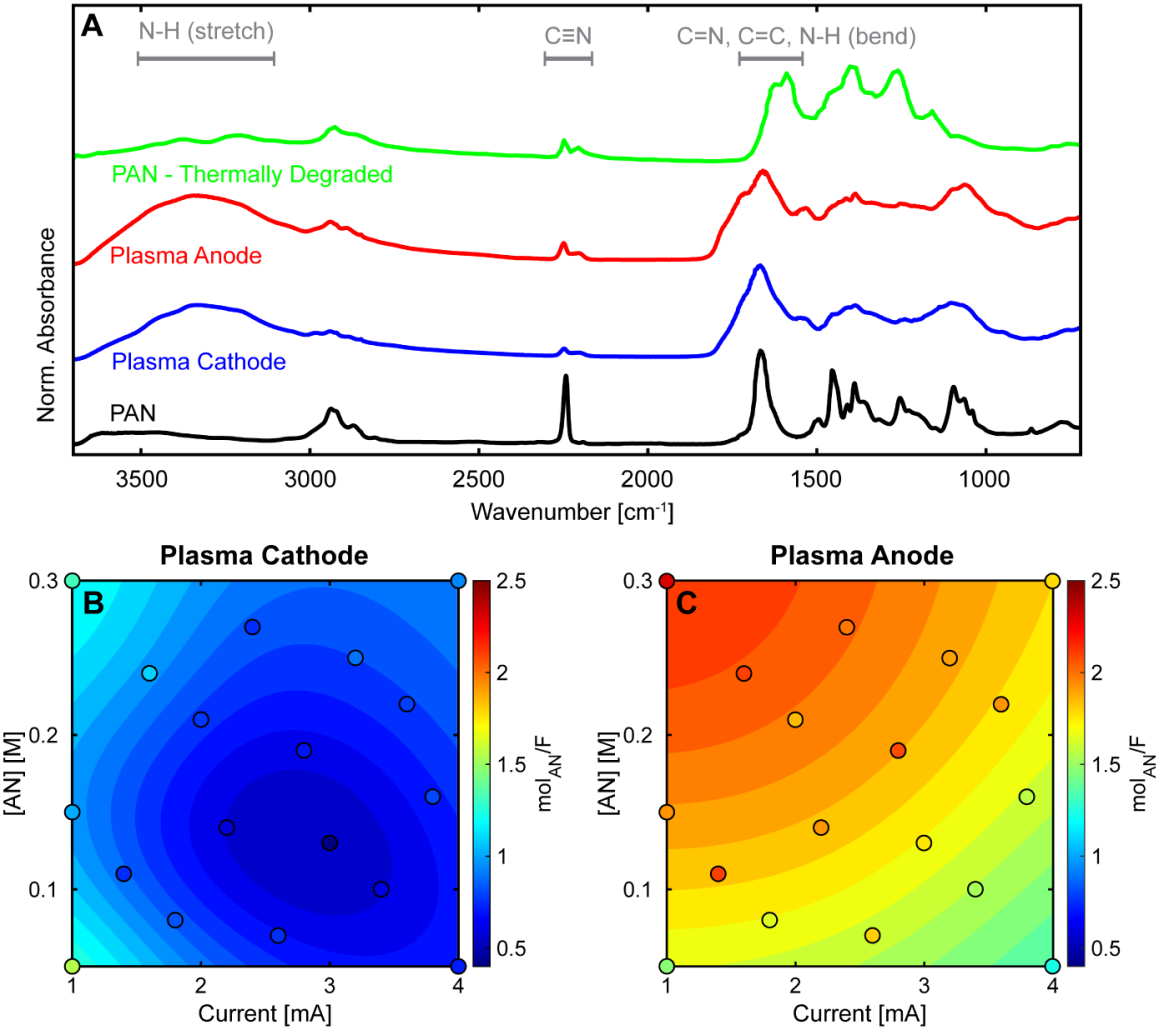
Chemical characterization and yields of plasma-produced polyacrylonitrile (PAN). (A) ATR-FTIR spectra comparing commercial PAN, plasma-derived solids from acrylonitrile, and thermally degraded PAN. Spectral features are consistent between plasma cathode and anode and with thermally degraded PAN. (B,C) PAN yield (mol_AN_/F) under plasma cathode (B) and plasma anode (C) conditions. The anode configuration increased solid product yields by 30–235% compared to cathode configuration. Surface plots show predicted values from data-driven surrogate models. The reaction solutions consisted of AN in DI water, and the reaction time was 15 min. Solid products were characterized using ATR-FTIR and quantified using thermogravimetric analysis (TGA).

The plasma-produced solids were characterized by FTIR spectroscopy (Figure 6A). The spectra showed characteristic vibrations expected in polymeric solids and bonds likely to form from AN polymerization. A key feature is the nitrile absorption peak around 2250 cm^–1^. Compared to pure PAN, the plasma-derived solid exhibits distinct modifications: the C≡N peak shows splitting and broadening, accompanied by an increase in the C=N peak (1600–1700 cm^–1^). These spectral changes suggest plasma-induced modifications of the polymer structure, likely including cross-linking and/or dehydrogenation of the polymer chains. Additionally, the presence of N–H bonds, indicated by a broad band at ∼ 3300 cm^–1^ and shoulders (1550–1650 cm^–1^) on the C=N absorbance, distinguishes the plasma-produced material from pure PAN. The broadening of vibrational bands relative to pure PAN indicates greater structural heterogeneity in the plasma-produced polymer. These trends mirror those seen in the thermal degradation of PAN, where higher temperatures induce cyclization and aromatization.^63^

Quantitative analysis of the solid yields (Figure 6B,C), expressed as moles of AN in the solid product per Faraday (mol_AN_/F) and calculated assuming PAN-equivalent composition, reveals striking differences between plasma configurations. With the plasma cathode (Figure 6B), solid yields generally remain below 1 mol_AN_/F, except at currents below 2 mA, where they increase to 1.25–1.5 mol_AN_/F. In contrast, the plasma anode (Figure 6C) produces consistently higher yields, exceeding 1.5 mol_AN_/F across conditions and reaching above 2 mol_AN_/F at low current (1 mA) and high AN concentration (0.3 M).

### Understanding the Balance between Faradaic and Non-Faradaic Processes in Plasma Electrochemistry

**Figure 7:**
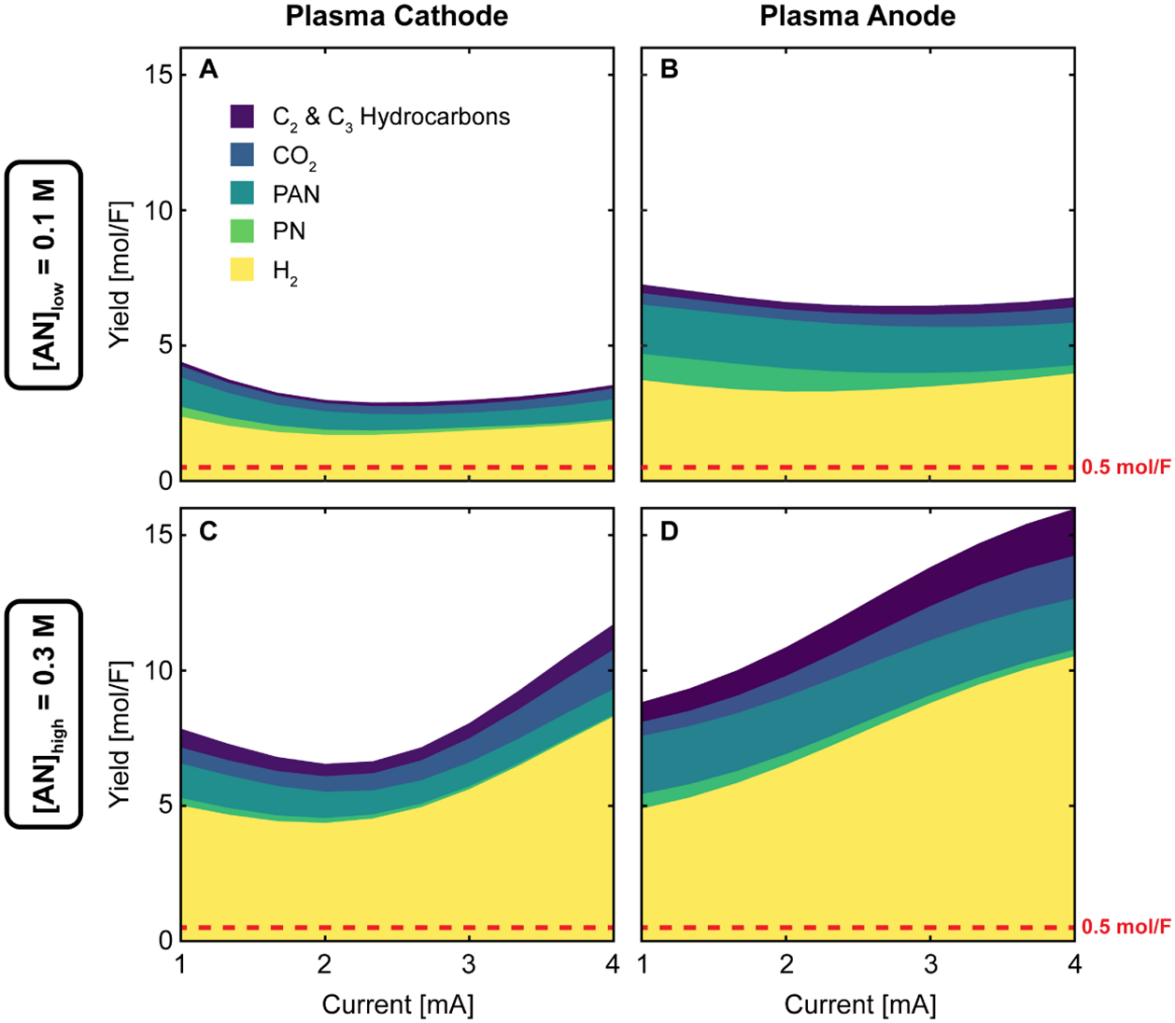
Product distribution from plasma electrochemical reactions of acrylonitrile (AN) at different operating conditions. (A,B) Low AN concentration ([AN]_low_ = 0.1 M) and (C,D) high AN concentration ([AN]_high_ = 0.3 M) using plasma cathode (A,C) and plasma anode (B,D) configurations. Products include solid polyacrylonitrile (PAN), liquid propionitrile (PN), and gases (H_2_, CO_2_, C_2_ and C_3_ hydrocarbons). Molar product yields (mol/F) exceed the theoretical maximum for charge transfer processes (0.5 mol/F, dashed horizontal line) by 5–32 times, indicating the dominance of non-Faradaic reactions. Plasma anode configuration produced higher total yields than plasma cathode across all conditions. Increasing AN concentration enhanced all product yields except PN, which decreased. The reaction solutions consisted of AN in DI water, and the reaction time was 15 min. Products were quantified using gas chromatography–mass spectrometry (GC-MS) for organic liquids, gas chromatography (GC) for gases, and thermogravimetric analysis (TGA) for solids.

We conducted experiments using a solvated electron scavenger to indirectly probe the contributions of charge transfer processes in plasma electrochemistry with AN. Surprisingly, the scavenger did not decrease the yields of potential charge transfer products (PN and H_2_); instead, PN yields increased with the plasma cathode while H_2_ production remained largely unchanged (Figure S1). We then quantified the total product distribution across solid (PAN), liquid (PN), and gas products (H_2_, CO_2_, C_2_, i.e., ethane/ethylene/acetylene, and C_3_, i.e., propane/propylene) at low (0.1 M) and high (0.3 M) AN concentration for both plasma configurations (Figure 7). For reference, conventional two-electron transfer products such as H_2_ and PN have a theoretical maximum yield of 0.5 mol/F (1 equiv./F), providing a clear threshold to identify non-charge transfer processes.

The product yields shown in Figure 7 dramatically exceeded charge transfer limits across all conditions, with the most striking result being a maximum total yield of 16 mol/F—32 times higher than possible via charge-transfer alone—observed with the plasma anode at high AN concentration (0.3 M) and high current (4 mA). The strong influence of plasma polarity on product distribution stems not from the direction of charge transfer but from the substantial energy difference between species reaching the liquid surface—electrons (<10 eV) in negative polarity versus argon ions (10–100 eV) in positive polarity. This energetic difference explains why the plasma anode configuration, with its more energetic ion bombardment, consistently produces higher yields across multiple products. The dominance of non-Faradaic processes becomes particularly evident at high [AN] and high current, where we observe a shift in reaction pathways: increased formation of degradation products (CO_2_ and C_2_/C_3_ hydrocarbons) alongside higher hydrogen yields suggests a transition from polymerization to dehydrogenation—behavior that parallels the thermal degradation of polyacrylonitrile.

**Figure 8:**
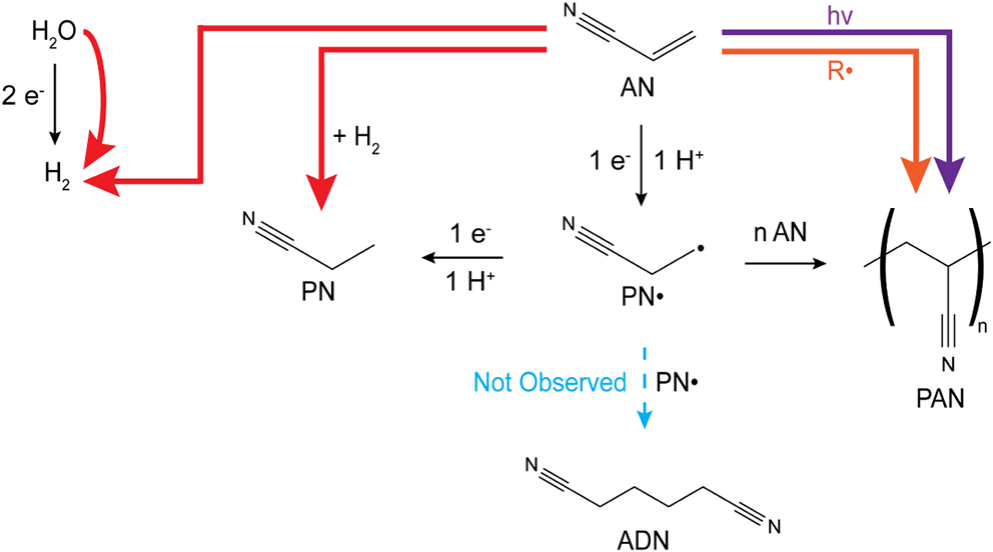
Proposed reaction mechanisms in plasma electrochemistry of acrylonitrile (AN). Faradaic (charge transfer) pathways (black arrows) show conventional electrochemical routes. No adiponitrile (ADN) formation pathway is included, as ADN was not detected under any conditions tested. Multiple polymerization mechanisms are proposed, including photopolymerization, free radical polymerization, and electrochemical polymerization. Propionitrile formation occurs through both conventional charge transfer and thermal hydrogenation using plasma-generated H_2_. Hydrogen generation proceeds via Faradaic and non-Faradaic pathways, with water and/or AN as reactants.

Based on the observed non-Faradaic yields and product distributions, we propose an integrated framework of reaction pathways at the plasma-liquid interface (Figure 8). While charge transfer processes can produce hydrogen gas, propionitrile, oligomers, and polymers, our results indicate that non-Faradaic mechanisms predominate.

Hydrogen generation in plasma electrochemistry can occur through both Faradaic and non-Faradaic mechanisms. While charge transfer contributes to hydrogen production, our results with water alone demonstrate that this contribution is relatively small. Most of the hydrogen is produced through non-Faradaic mechanisms such as ionization, excitation, or dissociation of water into hydrogen and hydroxyl radicals, which can recombine to form hydrogen gas and hydrogen peroxide. When AN is present, the contribution of charge transfer becomes even less significant, as evidenced by the dramatically higher yields (up to 16.6 and 21.0 equiv./F for plasma cathode and anode, respectively) that far exceed charge transfer limitations. In these conditions, AN dehydrogenation becomes a significant source of hydrogen, either directly producing hydrogen gas and AN dehydrogenation products or interacting with water to yield hydrogen gas, carbon dioxide, and various carbon products (e.g., C_2_ and C_3_ hydrocarbons). The relatively modest difference in hydrogen production between anode and cathode configurations, rather than an order of magnitude difference, suggests these processes likely share a common origin, possibly occurring in the gas phase.

Propionitrile formation similarly involves both Faradaic and non-Faradaic pathways. With the plasma cathode, yields approaching but not exceeding 1 equiv./F initially suggest a conventional electron-transfer mechanism. However, the plasma anode results challenge this interpretation, producing yields up to 2 equiv./F despite the absence of a known oxidative pathway from AN to PN. The presence of a noncharge transfer pathway for the plasma anode suggests that both Faradaic and non-Faradaic processes contribute to PN formation, even when yields appear consistent with conventional electrochemistry. We propose that the primary pathway involves plasma-induced hydrogenation, where active hydrogen species generated at the plasma-liquid interface react with AN to produce PN.

Polyacrylonitrile formation reveals perhaps the most striking departure from conventional electrochemistry, where PAN is not typically produced under equivalent conditions. While electrochemical polymerization could contribute to PAN formation in our system, the high yields (exceeding 1.5 mol_AN_/F with the plasma anode) and distinct FTIR spectral features indicate that non-Faradaic mechanisms dominate. These pathways could include photopolymerization initiated by the intense light emission from the plasma and free-radical polymerization driven by the highly reactive species at the plasma-liquid interface. The structural modifications of the AN-derived polymer observed by FTIR parallel thermal degradation pathways and further support the dominance of non-Faradaic processes in polymer evolution.

## Conclusions

Our systematic investigation of acrylonitrile reactions at the plasma-liquid interface reveals fundamental differences between plasma electrochemistry and conventional electrochemical systems, particularly in the dominance of non-Faradaic processes that lead to significantly different product distributions and yields. Several key findings support this conclusion. First, the absence of adiponitrile formation under reductive conditions (plasma cathode) and the substantial production of polyacrylonitrile and propionitrile under oxidative conditions (plasma anode) indicate fundamentally different reaction pathways from conventional electrochemistry. Second, the observation of high non-Faradaic product yields, up to 32 times higher than charge transfer limits, provides strong evidence for the dominance of non-Faradaic processes. This is particularly pronounced in the plasma anode configuration, where high-energy ion bombardment enhances energy transfer to the liquid interface. Third, the negligible effect of solvated electron scavengers in altering product distributions implies that Faradaic processes play a limited role in the plasma electrochemistry of acrylonitrile.

Based on these observations, we propose a reaction framework encompassing energy-transfer, photochemical, and radical-mediated pathways that explain the observed product distributions and their dependence on plasma polarity, current, and reactant concentration, providing mechanistic insights that will inform the development of future plasma-based synthesis methods. These findings suggest that dominant reaction pathways in the plasma electrochemical conversion of acrylonitrile are activated by non-Faradaic processes rather than charge transfer mechanisms. This understanding implies that effectively harnessing plasma electrochemistry requires careful consideration of the various non-Faradaic mechanisms at play for each specific reaction. Rather than just attempting to replicate conventional electrochemical reactions, future applications should leverage the unique non-Faradaic processes that occur at plasma-liquid interfaces. This could enable novel reaction pathways inaccessible to conventional electrochemistry, particularly in cases where high-energy intermediates or radical species are desired. Furthermore, these results underscore the need to develop new analytical methods to identify and quantify the short-lived reactive species at the plasma-liquid interface, as these intermediates likely play a crucial role in determining reaction outcomes. Our findings provide a fundamental framework for understanding and optimizing plasma electrochemical processes, potentially establishing new routes for sustainable chemical manufacturing through plasma-driven synthesis.

## Supporting Information Available

Technical details including chemicals and materials, reactor configuration, characterization (solid/liquid/gas), data analysis, solvated electron scavenger experiments, optical emission spectroscopy, product quantification, and plasma operation (PDF)

## Abbreviations

AN: acrylonitrile
ADN: adiponitrile
PN: propionitrile
PAN: polyacrylonitrile
GPR: Gaussian process regression
GC-MS: gas chromatography–mass spectrometry
FTIR: Fourier transform infrared spectroscopy
UV/VUV: ultraviolet/vacuum ultraviolet
TGA: thermogravimetric analysis
