# Supplementary material for: Exploring the Balance between Faradaic and Non-Faradaic Processes in Organic Chemical Reactions at Plasma-Liquid Interfaces
Source: J Am Chem Soc. 2025 Apr 12;147(16):13937–47. doi: 10.1021/jacs.5c02740 (PMC12023021; doi:10.1021/jacs.5c02740)
Supplement: Supplementary file 1 — ja5c02740_si_001.pdf [file ja5c02740_si_001.pdf]

Supporting Information:

Exploring the Balance Between Faradaic and non-Faradaic Processes in Organic Chemical Reactions at Plasma-Liquid Interfaces

Casey K. Bloomquist,[a] Daniel Naumov,[a] Ahrin Yang,[a] Ricardo Mathison,[a] Benjamin D. Herzog,[b] William J. Tenn III,[b] Miguel A. Modestino[a,*] and Eray S. Aydil[a,*]

[a]New York University, Tandon School of Engineering, Department of Chemical and Biomolecular Engineering, 6 Metrotech Center, Brooklyn, NY 11201, USA

[b]INVISTA, Texas Technology Center, 21920 Merchants Way, Katy, TX 77450 USA

E-mail: modestino@nyu.edu, aydil@nyu.edu

Table of Contents

Experimental Methods 2

Solvated Electron Scavenger Experiments 3

Optical Emission Spectroscopy 4

Product Quantification 6

Plasma Operation 9

References 14

Experimental Methods

Materials. All chemicals were purchased from commercial sources and used as received. Acrylonitrile (AN, 99%), propionitrile (PN, 99%), polyacrylonitrile (PAN, average MW 150,000), and heptane (99%) were obtained from Sigma-Aldrich. Toluene (99.9%) was purchased from VWR. N,N-Dimethylformamide (DMF, 99%) was purchased from Alfa Aesar. Hydrogen gas standards were obtained from GASCO (310 ppm in air) and DOD Technologies (1000 ppm in air). A standard gas hydrocarbon mixture containing 1% butane (99.99%), 1% ethylene (99.99%), 1% ethane (99.99%), 1% propane (99.99%), 1% propylene (99.95%), 1% CO2 (99.999%) and 1% CO (99.99%) in nitrogen (99.999%) was purchased from Advanced Specialty Gases for gas chromatography calibration. Ultra-high purity argon (99.999%) was obtained from AirGas.

Plasma Electrochemical Setup. Experiments were conducted in custom H-cell reactors fabricated using a Stratasys Objet30 3D printer with VeroClear resin. The plasma electrode consisted of a stainless steel needle (0.5 mm diameter, Roboz Surgical Instrument Co.) crimped within a copper tube (McMaster, Super-Conductive 101 Copper, 1/8" OD). Argon gas flow (60.7 sccm) was controlled using a Brooks Instrument GF40 mass flow controller. The counter electrode was a platinum mesh (Thermo Fisher Scientific) immersed in deionized water (1-2 µs/cm). The two chambers were separated by either a Nafion N-117 or Aquivion E98-15S ion exchange membrane. A positive or negative DC voltage (±2.5 kV) was applied using a Stanford Research Systems PS325 power supply in series with a 160 kΩ ballast resistor (Clarostat RW47V164) to maintain plasma currents of 1-5 mA.

Reaction Conditions. The plasma-side solution consisted of AN (0-0.3 M) in 5 mL of deionized water. The plasma needle was positioned 1-3 mm above the solution surface. All experiments were conducted at room temperature for 15 minutes with no stirring.

Solid Product Analysis. Solid products were collected and characterized using FTIR spectroscopy and thermogravimetric analysis (TGA). Solid samples were dissolved in DMF for FTIR characterization and analyzed using a Thermo Scientific Nicolet iS50 FT-IR spectrometer equipped with a multibounce ZnSe polished 45° trapezoidal ATR crystal from REFLEX Analytical Corporation. Spectra were collected after solvent evaporation at 40-50°C. Multiple depositions were performed to achieve adequate signal strength. Mass quantification was performed using a TA Instruments TGA 550. Samples were prepared by depositing a 20 µL aliquot of the DMF solution into 100 µL platinum pans (TA Instruments) and heating at 40°C for 15 minutes. This process was repeated 5 times to achieve sufficient sample mass while preventing solvent overflow. TGA analysis was performed using nitrogen gas and the following temperature program: Equilibrate 50 °C; Isothermal 2.0 min; Ramp 20 °C/min to 600 °C; Isothermal 15 min; Equilibrate 50 °C.

Liquid Phase Analysis. Organic products were extracted using liquid-liquid extraction with toluene (2:1 sample:toluene). The organic phase was analyzed using a Shimadzu QP2010/GCMS-QP2020 NX gas chromatograph-mass spectrometer equipped with a Rxi-5Sil MS GC Capillary Column (30 m, 0.25 mm ID). The temperature program was 40 °C for 1 min, ramp 20 °C/min to 300 °C, hold 300 °C for 10 min. Components were quantified using calibration curves generated with heptane as an internal standard. Hydrogen peroxide was quantified using colorimetry using titanium (IV) oxysulfate ($TiOSO_4$).[1] Reaction solutions were mixed in a 10:1 ratio with TiOSO4, and the absorbance at 410 nm was measured using an Agilent Cary 60 UV-Vis. Quantification was performed using a calibration curve of known concentrations. Solutions that saturated the detector were diluted before mixing with TiOSO4.

Gas Phase Analysis. Gaseous products were collected in Tedlar sample bags and analyzed using an Agilent Micro GC 990 with a MolSieve 5A column (channel 1) and PoraPLOTQ column (channel 2). Acquisition parameters for channel 1 were injection temperature 110 °C, injection time 80 ms, column temperature 80 °C, carrier gas Ar, run time 90 s. Acquisition parameters for channel 2 were injection temperature 110 °C, injection time 200 ms, column temperature 70 °C, carrier gas He, run time 90 s. Quantification was performed using calibration curves generated from standard gas mixtures.

Data Analysis and Modeling. Experimental conditions were selected using the Hammersley quasi-random sequence algorithm (author: Mansour Torabi) implemented in MATLAB R2024a. This low-discrepancy sampling method generated uniformly distributed points across a two-dimensional parameter space defined by current (1 – 4 mA) and AN concentration (0.05 – 0.3 M). The experimental conditions were used for both plasma cathode and plasma anode experiments. Gaussian Process Regression (GPR) models were constructed using fitrgp in MATLAB's Statistics and Machine Learning Toolbox. The models were trained using the following input arguments: KernelFunction = "matern52", FitMethod = "exact," PredictMethod = "exact," Standardize = 1. The noise standard deviation parameter, Sigma, was held constant (ConstantSigma = True) and set to a value consistent with experimental errors. Model validation was conducted by comparing the GPR-predicted standard deviations at each point with experimental measurement uncertainties. The trained models were used to generate continuous predictions across the parameter space by using the predict function to evaluate the GPR on a regular grid of points.

Solvated Electron Scavenger Experiments

Previous research has used scavenger experiments to investigate the impact of solvated electrons on reaction mechanisms in plasma-liquid systems.[2–5] These experiments use compounds that selectively react with, or "scavenge," specific reactive species, such as solvated electrons. By observing how a solvated electron scavenger affects product formation, researchers can infer the role of solvated electrons, and thus charge transfer, in the overall process. We performed scavenger experiments with AN in 0.5 M sodium nitrate ($NaNO_3$, Sigma Aldrich ReagentPlus® ≥ 99%), a solvated electron scavenger used in several previous investigations.[2–5] To control for changes in solution conductivity and plasma characteristics due to salt addition, we also conducted the same experiments with 0.5 M sodium perchlorate ($NaClO_4$, Sigma Aldrich ACS Reagent ≥ 98%), an inert salt that reportedly does not readily react with solvated electrons.[2,3] By matching the solution conductivity with $NaNO_3$ or $NaClO_4$, we aimed to create comparable plasma conditions where the only difference is the presence or absence of a solvated electron scavenger.

Figure S1 shows the results of the scavenger experiments for negative and positive polarity with 0.5 M $NaClO_4$ and 0.5 M $NaNO_3$. Unexpected trends emerged when comparing the effects of the scavenger ($NaNO_3$) with the inert salt ($NaClO_4$). Contrary to expectations, the scavenger appeared to increase PN yield for the plasma cathode while having a negligible impact on H_2 production. In the plasma anode configuration, the scavenger did not significantly affect PN or H_2 yields compared to the inert salt, consistent with the injection of positive argon ions rather than electrons in anodic plasma. However, it is important to note that scavenger experiments may have limitations in plasma systems. The very high overpotentials present in our plasma setup could enable various unexpected reactions involving the added salts (such as the reduction of sodium, nitrate, or perchlorate ions and the subsequent reactions of their reduction products with liquid-phase species), potentially affecting the performance of the scavenger itself.

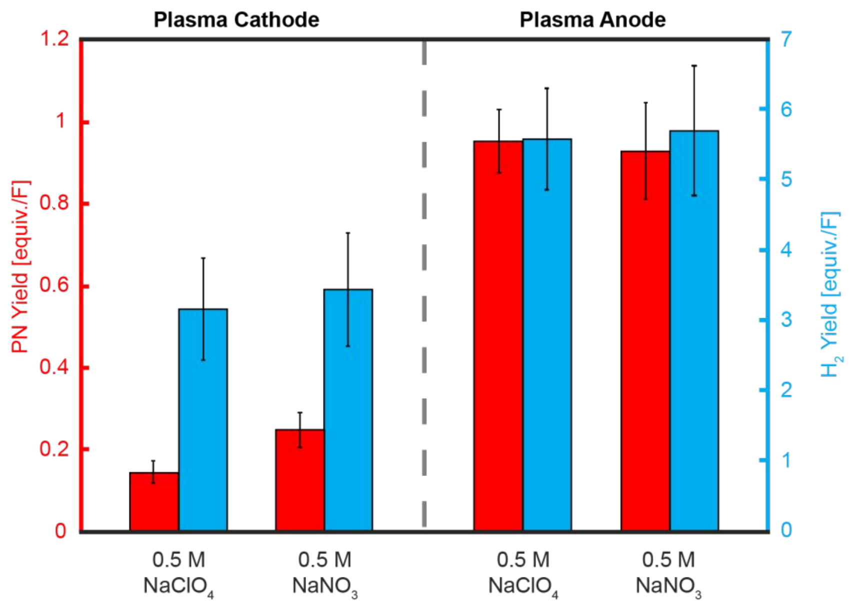

Figure S1. Scavenger experiment results. PN and H_2 yields in $NaClO_4$ (control) and $NaNO_3$ (solvated electron scavenger) for plasma cathode and anode. The conductivity of the $NaClO_4$ and $NaNO_3$ were matched at 0.5 M to achieve similar plasma conditions. All experiments were performed at 2 mA plasma current and 0.1 M AN concentration for 15 minutes.

Optical Emission Spectroscopy

We employed optical emission spectroscopy (OES) to complement our product analysis and characterize the plasma species present during the reaction (Figure S2). Measurements were collected using an Ocean Optics USB 2000 spectrometer coupled to an Ocean Optics P600-2-SR fiber optic cable, which was directed at the plasma through a quartz window. We optimized the integration time to prevent peak saturation and began collecting spectra immediately after plasma initiation. The OES spectra exhibited temporal variations, with signal intensity gradually diminishing due to water vapor condensation on the window. Figure S2 presents representative OES spectra with labeled peaks for two conditions: the plasma anode operating at 4 mA with 0.3 M AN and a reference spectrum using DI water.

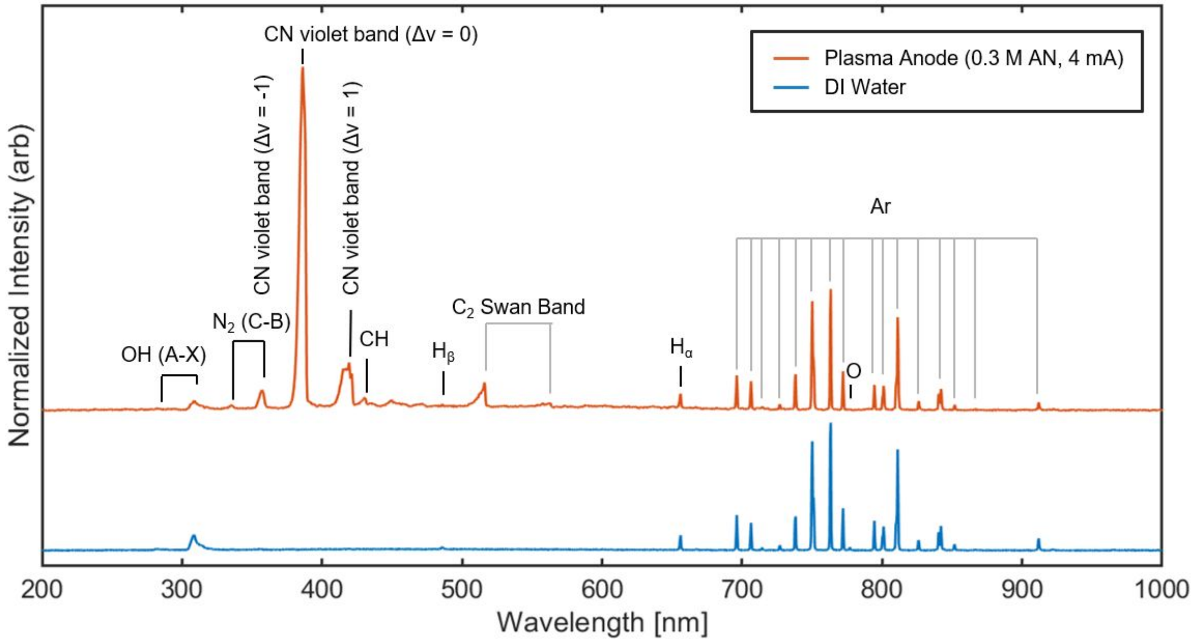

Figure S2. Optical emission spectra of the plasma anode operating at 4 mA with 0.3 M acrylonitrile (AN) solution and deionized (DI) water. The AN-containing solution exhibits peaks corresponding to OH(A-X), CN violet band, CH, C_2 Swan band, H_α, H_β, O and Ar transitions. In contrast, the DI water spectrum shows only OH (A-X), H_α, H_β, O, and Ar emissions.

We compared the intensity trends of CN, C_2, OH, and H species at low (0.05 M) and high (0.3 M) AN concentration, low (1 mA) and high (4 mA) plasma currents, and between plasma cathode and anode configurations, shown in Figure S3. CN emissions, indicative of AN dehydrogenation, intensify with increasing current or concentration and are more pronounced in positive polarity. C2 species are negligible at low AN concentrations but become significant at high concentrations, with the highest intensities observed at high current/high concentration conditions. OH emissions, primarily from water decomposition, decrease with increasing concentration, suggesting preferential plasma-AN interactions over plasma-water reactions. Similarly, H emissions increase with current but decrease with AN concentration, further supporting the preferential plasma-AN reactions at high AN concentration.

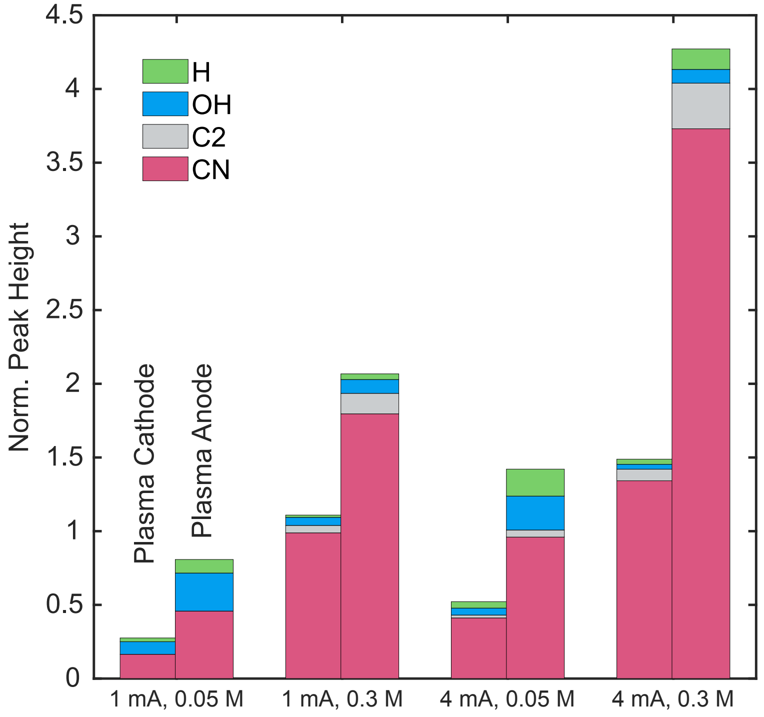

Figure S3. Emission intensity trends of CN, C_2, OH, and H species as a function of experimental conditions: AN concentrations (0.05 M and 0.3 M), plasma currents (1 mA and 4 mA), and plasma polarity (anode versus cathode). All species exhibit higher peak intensities with the plasma anode compared to the cathode configuration. CN and C_2 emissions show particularly dramatic intensity increases at high current (4 mA) and high AN concentration (0.3 M).

Product Quantification

The solid mass is quantified in moles of AN per Faraday calculated by,

$$mol_{AN}/F = \frac{\frac{m_{solid}}{M_{AN}}}{it/_{F}} (S1)$$

where the m_{solid} is the solid mass determined from TGA (Figure S4), M_{AN} is the molar mass of AN, i is the current, t is experimental time, and F is Faraday's constant (96,485 C/mol).

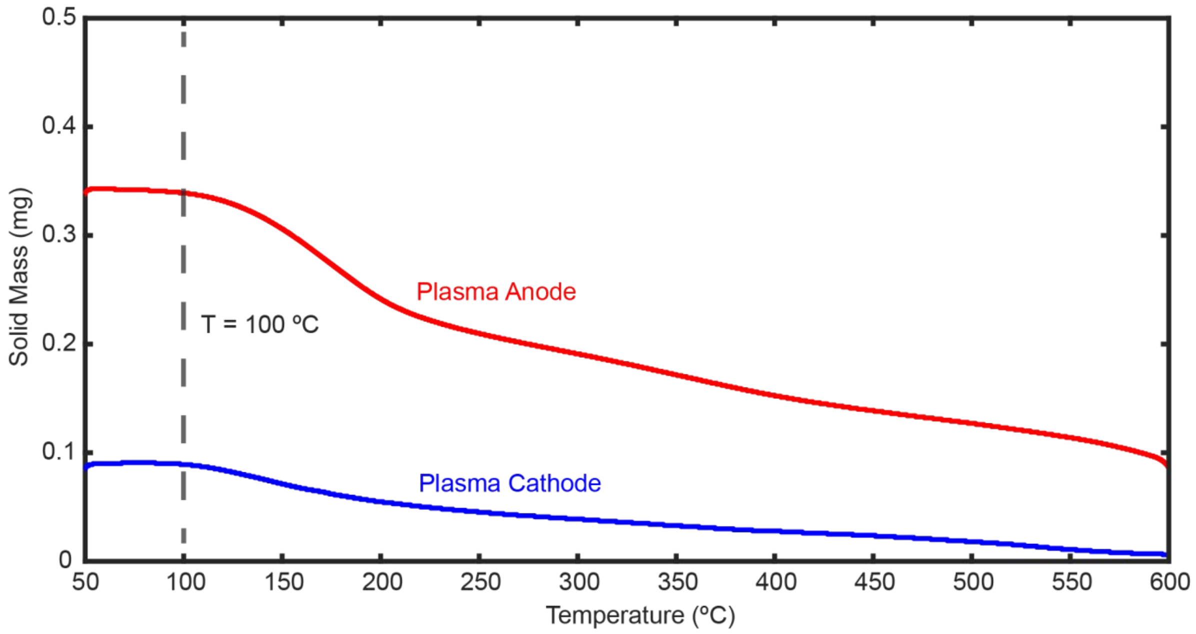

Figure S4. Example Thermogravimetric Analysis (TGA) data for plasma cathode and anode for experimental conditions of 4 mA, 0.2 M AN. Solid mass was determined by the mass at 100 °C. Mass loss at higher temperatures is due to further thermal degradation of the solid.

The liquid product (PN) is quantified in equivalents per Faraday (equiv./F) calculated by,

$$PN\ equiv./F = \frac{C_{PN}V}{it/_{nF}} (S1)$$

where C_{PN} is the PN concentration determined from GCMS (Figure S5), V is the experimental volume (5 mL), i is the current, t is experimental time, n is the number of electrons involved in the reaction (2 for PN) and F is Faraday's constant (96,485 C/mol).

Hydrogen gas is quantified in equivalents per Faraday (equiv./F) calculated by,

$$H_2\ equiv./F = \frac{C_{H_2}(ppm)\,\frac{PV}{RT}}{it/_{nF}} (S1)$$

where $C_{H_2}(ppm)$ is the H_2 concentration in ppm determined from GC (Figure S5), P is atmospheric pressure, V is the experimental volume determined from gas flowrate and experimental runtime, R is the ideal gas constant, T is the temperature, i is the current, t is experimental time, n is the number of electrons involved in the reaction (2 for H_2) and F is Faraday's constant (96,485 C/mol). Other gases (CO_2, C_2 & C_3 hydrocarbons) were quantified in a similar fashion to H_2 but in in moles per Faraday using a mixed hydrocarbon standard gas for calibration.

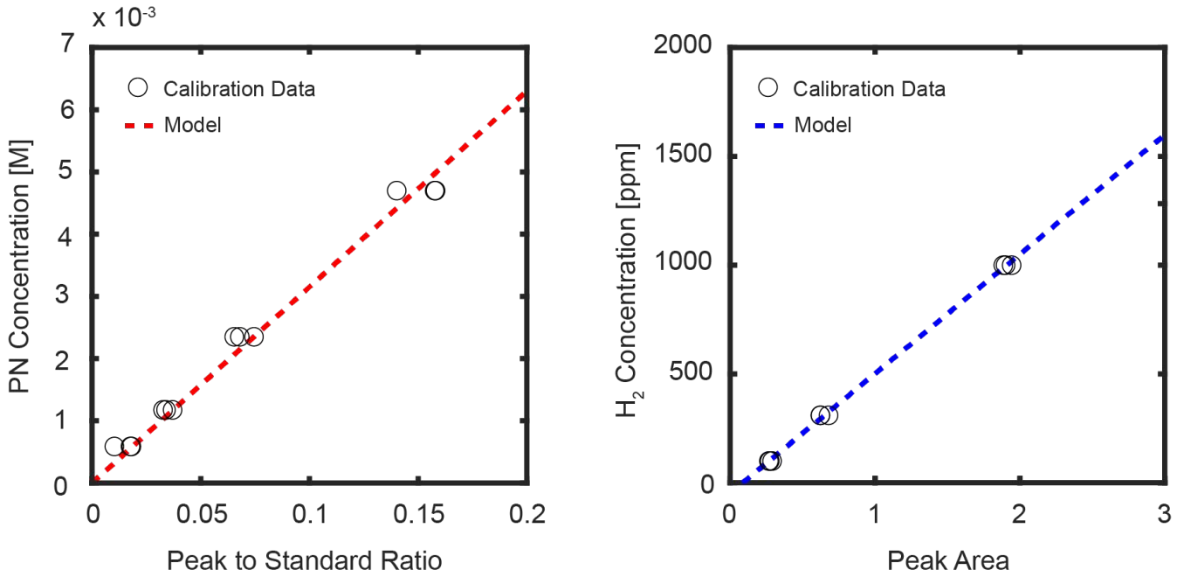

Figure S5. GC calibration models for propionitrile (PN) and hydrogen gas (H_2). PN concentrations are determined by relating the PN to standard peak ratio to known concentrations. H_2 concentrations are determined by relating peak areas to known concentrations using gas standards. Three samples were measured for each concentration.

Adiponitrile (ADN) was not detected under any experimental conditions. We established a detection limit of 0.5 mM ADN with GC/MS, as described above. Figure S6 illustrates the ADN peak in GC/MS for 1 mM and 0.5 mM ADN standards, alongside a post-plasma sample (2.5 mA, 0.15 M AN, negative polarity, 30 minutes). Under these conditions, 100% Faradaic efficiency toward ADN would produce a concentration of ~5 mM, meaning our method could detect ADN at Faradaic efficiencies as low as 10%.

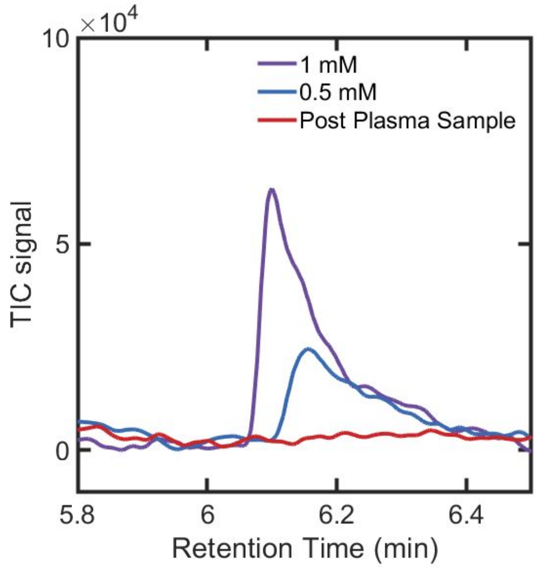

Figure S6. Comparison of GC/MS chromatograms showing the detection limit for adiponitrile (ADN) standards (1 mM and 0.5 mM) and a post-plasma sample conducted at 2.5 mA, 0.15 M AN, negative polarity for 30 minutes.

Plasma Operation

We attempted to operate the plasma electrosynthesis system under conditions comparable to conventional electrosynthesis, though some differences were unavoidable. Conventional ADN electrosynthesis typically uses electrolytes containing phosphate ions (Na_3PO_4) for enhanced conductivity and pH buffering, tetraalkylammonium salts for improved organic reactant solubility, and ethylenediaminetetraacetic acid (EDTA) as a chelating agent. These additives were either unnecessary for plasma electrosynthesis or led to undesired effects (e.g., salt precipitation). We used deionized (DI) water without additives, simplifying the electrolyte while still achieving current densities (~100 mA cm^{-2}) comparable to conventional ADN electrosynthesis. However, controlling the current density of the plasma system proved challenging as we could only set the current directly. The current density was then determined by the set current and the plasma diameter. Notably, the plasma diameter is a function of both the current and the solution conductivity. Figures S7 and S8 show images of the plasma cathode and plasma anode, respectively, captured immediately after plasma initialization at various currents and AN concentrations. However, as shown in Figure S9, the plasma area, and thus the plasma current density, contracted over time during an experiment, leading to a range of estimated current densities from 25 mA/cm^{-2} to >1000 mA/cm^{-2}. Additionally, the total current in the plasma system (1 – 4 mA) was initially considerably lower than in conventional electrosynthesis (typically 100s of mA, depending on electrode area).

Figure S10 shows the potential and current behavior versus time for an example plasma experiment (4 mA, 0.3 M AN). After an initial spike after initiation, the potential reached steady operation. The plasma anode potential tended to be more steady than the plasma cathode, which saw larger variations as shown in Figure S9A. Even with variations in potential, the plasma current was maintained at the set current (4 mA as shown in Figure S9B).

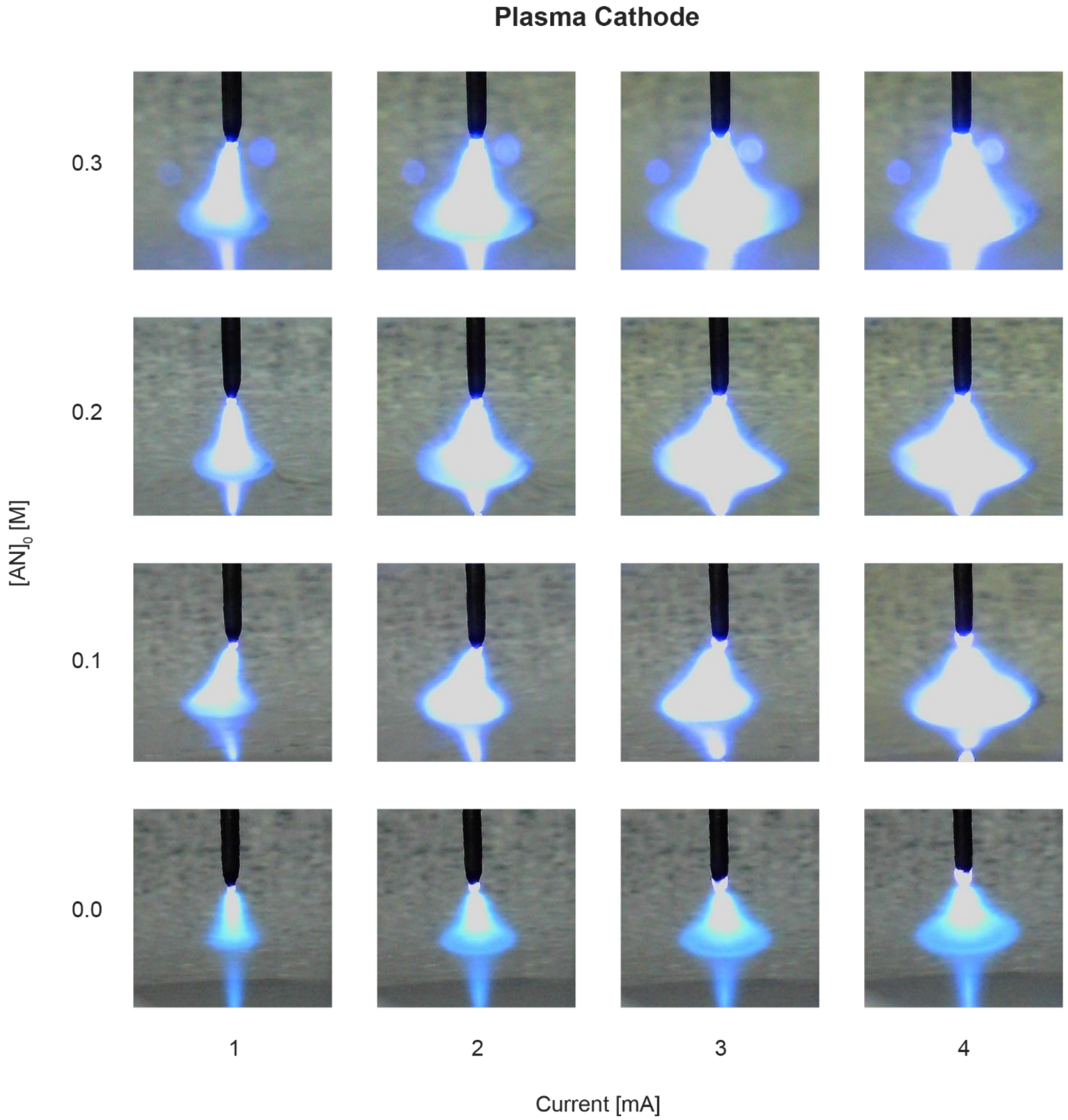

Figure S7. Plasma cathode images as a function of current (1-4 mA) and AN concentration (0.0-0.3 M) captured immediately after plasma initialization. Both increasing current and AN concentration resulted in larger plasma areas.

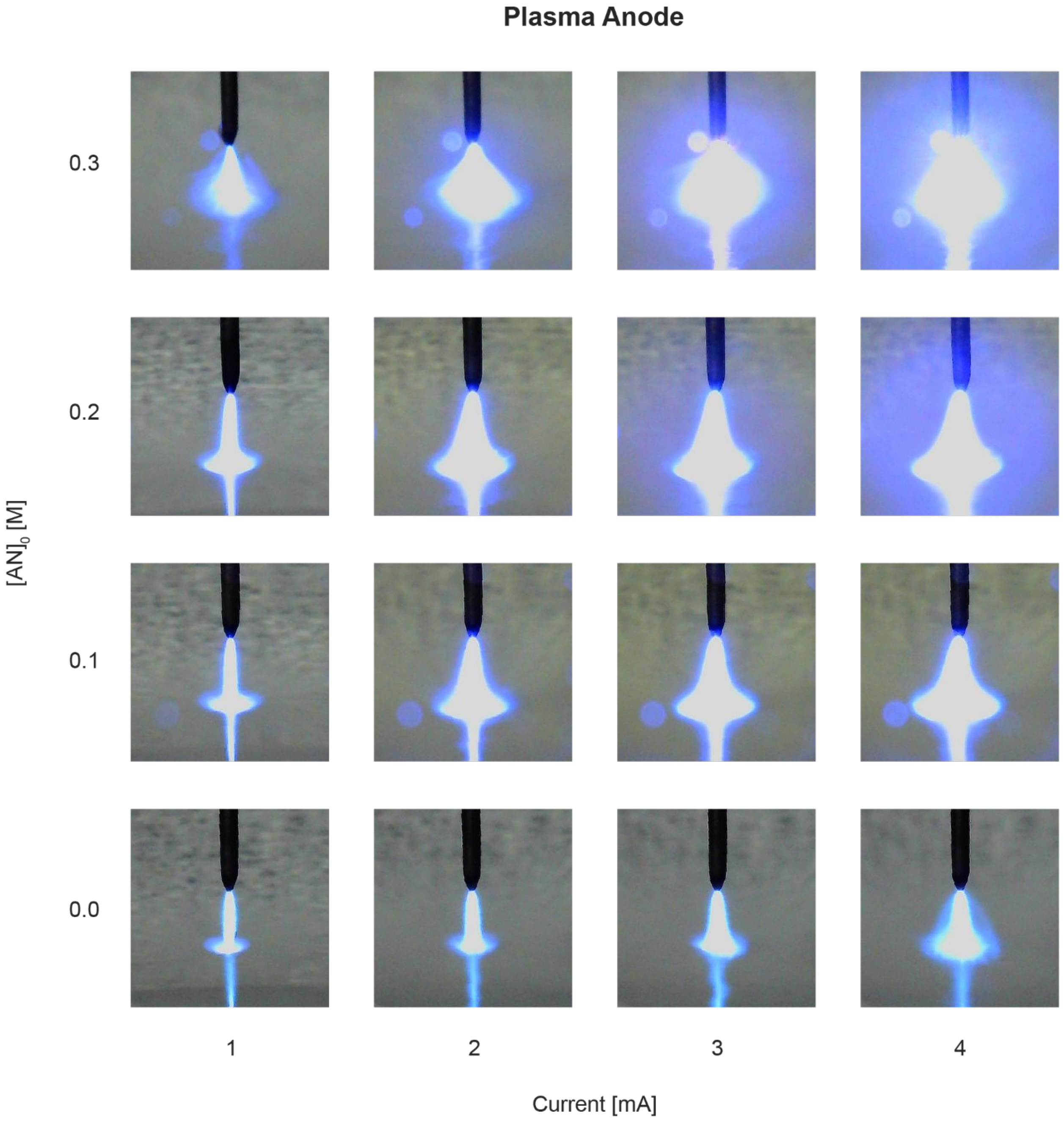

Figure S8. Plasma anode images as a function of current (1-4 mA) and AN concentration (0.0-0.3 M) captured immediately after plasma initialization. Both increasing current and AN concentration resulted in larger plasma areas.

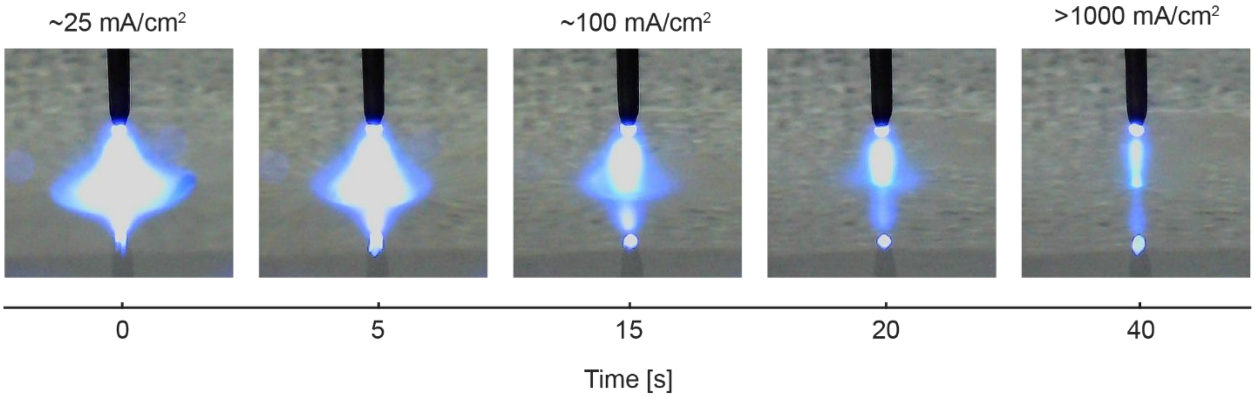

Figure S9. Temporal evolution of plasma current density measured under fixed experimental conditions: plasma cathode operation at 2 mA with 0.2 M acrylonitrile (AN) solution.

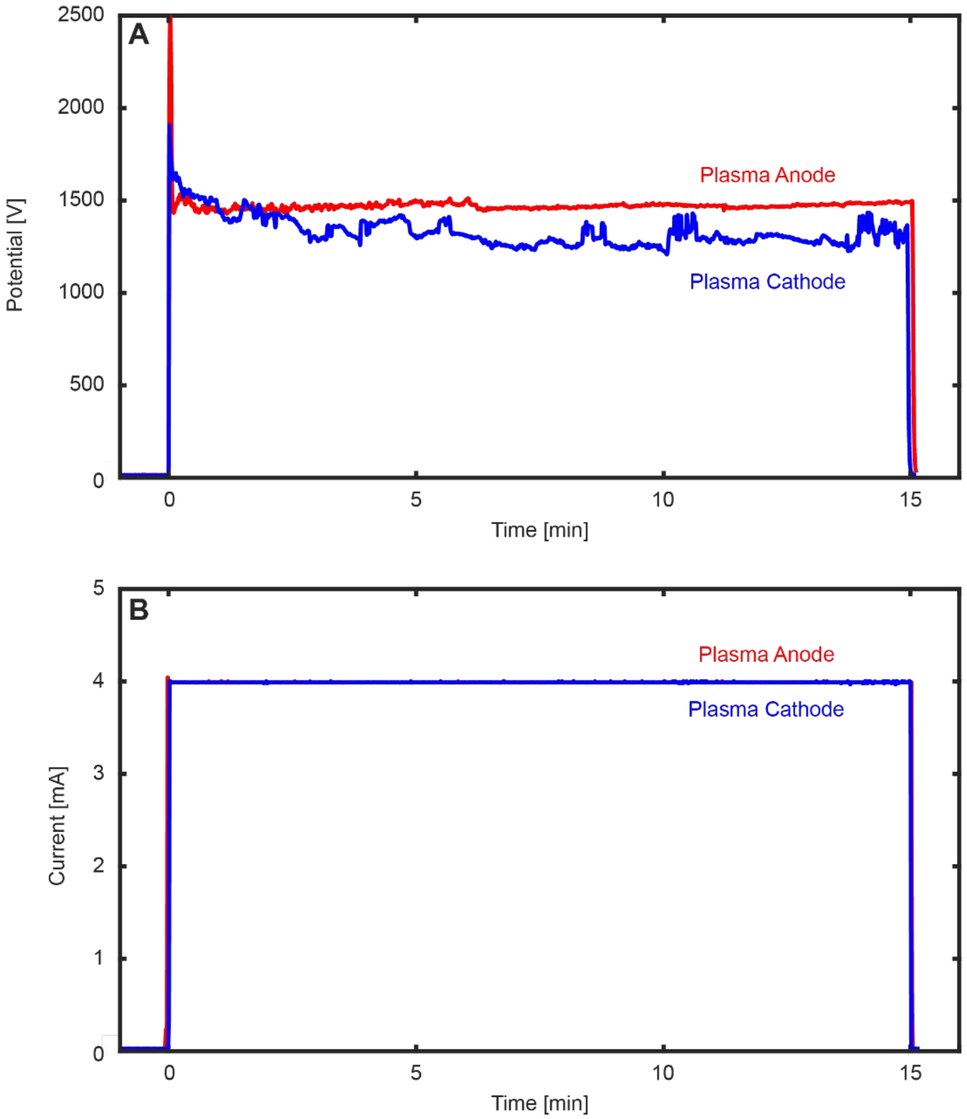

Figure S10. Time-dependent electrical characteristics of plasma electrodes operated at 4 mA and 0.3 M AN. (A) Potential versus time showing initial voltage spike followed by stable operation at ~1500 V for the plasma anode (red) and ~1300 V for the plasma cathode (blue). (B) Current versus time demonstrating constant 4 mA operation for both electrodes throughout the 15-minute experiment.

References

(1) Eisenberg, G. Colorimetric Determination of Hydrogen Peroxide. *Ind. Eng. Chem.* **1943**, *15* (5), 327–328.

(2) Rumbach, P.; Bartels, D. M.; Sankaran, R. M.; Go, D. B. The Solvation of Electrons by an Atmospheric-Pressure Plasma. *Nat. Commun.* **2015**, *6* (1), 7248. https://doi.org/10.1038/ncomms8248.

(3) Wolff, R. K.; Bronskill, M. J.; Hunt, J. W. Picosecond Pulse Radiolysis Studies. II. Reactions of Electrons with Concentrated Scavengers. *J. Chem. Phys.* **1970**, *53* (11), 4211–4215. https://doi.org/10.1063/1.1673923.

(4) Hawtof, R.; Ghosh, S.; Guarr, E.; Xu, C.; Mohan Sankaran, R.; Renner, J. N. Catalyst-Free, Highly Selective Synthesis of Ammonia from Nitrogen and Water by a Plasma Electrolytic System. *Sci. Adv.* **2019**, *5* (1), eaat5778. https://doi.org/10.1126/sciadv.aat5778.

(5) Wang, J.; Üner, N. B.; Dubowsky, S. E.; Confer, M. P.; Bhargava, R.; Sun, Y.; Zhou, Y.; Sankaran, R. M.; Moore, J. S. Plasma Electrochemistry for Carbon–Carbon Bond Formation via Pinacol Coupling. *J. Am. Chem. Soc.* **2023**, *145* (19), 10470–10474. https://doi.org/10.1021/jacs.3c01779.
